# Effects of open-channel blocking peptides in Na_V_1.5 *Δ*KPQ

**DOI:** 10.1016/j.bpj.2025.05.030

**Published:** 2025-06-02

**Authors:** Maria Riedersberger, Madalina Woltereck, Paul J. Wagner, Kristin Focke, Sarina Höller, Angelika Lampert, Christian Alzheimer, Stefan Düsterhöft, Tobias Huth

**Affiliations:** 1 Institut für Physiologie und Pathophysiologie, Friedrich-Alexander-Universität Erlangen-Nürnberg, Erlangen, Germany; 2 Institut für Neurophysiologie, Universitätsklinikum RWTH Aachen University, Aachen, Germany; 3 Institute of Molecular Pharmacology, Universitätsklinikum RWTH Aachen University, Aachen, Germany

## Abstract

Resurgent sodium currents (INaRs) result from an unorthodox gating behavior of voltage-activated sodium channels (Na_V_), allowing transient re-openings during repolarization from an apparently inactivated state. In both native cells not normally exhibiting INaR and in heterologous expression systems, intracellular delivery of small positively charged peptides through the recording pipette elicits robust INaRs, suggesting that INaRs arise from a peptide-mediated open-channel block that is relieved upon repolarization. Here we examined the hNa_V_1.5 *Δ*KPQ mutant, which causes a long QT syndrome in the heart, to probe the open-channel block hypothesis of INaR in a channel with altered inactivation properties due to the deletion near the canonical inactivation gate. We investigated INaRs with peptides derived from the hNa_V_*β*4 subunit, FGF13-1a and FGF14-1a, using the HEK293T expression system. Surprisingly, the peptides not only gave rise to pronounced INaRs with unusually fast kinetics but also altered the late sodium current of the channel mutant. To elucidate the molecular basis of these effects, we employed AlphaFold modeling of hNa_V_1.5, incorporating the *Δ*KPQ mutation and the *β*4 peptide. This model supports the open-channel block mechanism of INaR and its mutual exclusivity with fast inactivation. It also demonstrates a lack of interaction between the IFM linker and the C-terminal domain in hNa_V_1.5 *Δ*KPQ, offering a plausible explanation for why the peptides are capable of affecting both INaR and persistent currents (INaPs). Finally, the peptides generated a considerable increase in repolarization-associated Na^+^ currents with the *Δ*KPQ mutant, highlighting the presumed impact of pathologically enhanced INaR on cardiac electrophysiology.

## Significance

Voltage-activated sodium channels produce a transient current that initiates the cardiac action potential; incomplete inactivation of these channels generates a late current that shapes the plateau phase. The resurgent current that arises from open-channel block by an intracellular particle could contribute to the late current. We investigated the late current of the human Na_V_1.5 channel carrying the *Δ*KPQ mutation, which causes LQT3 syndrome, by applying different blocking particles. For the first time, we used AlphaFold to model how one of these particles docks at the channel’s internal mouth. Patch-clamp recordings, combined with our modeling, highlight the interplay between the blocking particle and the channel’s inactivation machinery and provide a mechanism by which the *Δ*KPQ mutation may amplify the late current.

## Introduction

The congenital long QT syndrome is characterized by the prolongation of the QT interval in the electrocardiogram. Affected individuals are at risk of potentially fatal ventricular arrhythmias. Prolongation of the QT interval results from delayed repolarization of the cardiac action potential (AP), reflecting an altered balance between depolarizing and repolarizing currents. The cardiac AP is initiated by the sodium channel, Na_V_1.5, which mediates a fast-activating transient inward current (INaT), followed by a small noninactivating late sodium current (1). Mutations in the gene encoding hNa_V_1.5 (*SCN5A*) that lead to increased late sodium currents—one of several possible mechanisms prolonging the cardiac AP—mediate additional calcium influx (2). The deletion mutant *Δ*KPQ was the first *SCN5A* variant to be linked to congenital long QT syndrome (3,4). The mutant *α* subunit lacks three amino acids (lysine, proline, and glutamine) at positions 1505–1507. Notably, the deletion is located intracellularly in the linker connecting domain III and IV. This region is crucial for fast inactivation, a distinct feature of voltage-activated sodium channels (Na_V_) (5). As the inactivation mechanism is impaired, the *Δ*KPQ mutation leads to an increased fraction of late sodium current during the plateau phase and myocyte repolarization (6,7,8,9). Late Na^+^ currents due to incomplete fast inactivation have also been observed in neurons, where they are referred to as persistent currents (INaP) (10,11).

In addition to INaT and INaP, a third manifestation of the sodium current, termed resurgent current (INaR), was first observed in cerebellar Purkinje cells (12) and subsequently in other neuronal cell types. Although arising from the same channel protein causing the upstroke of the AP, INaP and INaR are widely considered as Na^+^ current entities that are phenomenologically and mechanistically distinct in nature. Regarding the persistent current, incomplete inactivation beyond the “window” predicted by the overlap of the classical steady-state inactivation and activation curves enables INaP over a broad voltage range (13). The resurgent current is thought to arise from an alternative mode of fast inactivation, involving a separate blocking particle, that transiently blocks the open channel before the canonical mechanism of inactivation kicks in (14). Candidate open-channel blockers are positively charged parts of the Na^+^ channel *β*4 subunit or fibroblast growth factor 14 (FGF 14-1a), as the intracellular delivery of short peptides containing these parts induced INaR in cells not normally exhibiting this Na^+^ current manifestation (14,15). Upon repolarization, Na^+^ influx expels the blocking particle from the channel pore, allowing for a transient surge of inward current before conventional inactivation or deactivation take effect (16). In functional terms, INaR promotes fast firing, because unblocked Na^+^ channels are immediately available—they do not have to first recover from conventional inactivation. In addition, INaR provides a depolarizing current to shorten the inter-spike interval (17,18).

The current conceptual framework of INaR has limitations. Expression of full-length *β*4 subunit, together with Na^+^ channel *α* subunits, fails to produce INaR in heterologous systems (19,20,21). In contrast, heterologous FGF14-1a (FHF4A) expression did induce INaR when co-expressed with Na_V_1.8, Na_V_1.9, and to a lesser extent with Na_V_1.5, Na_V_1.7 (22). Knockdown of *β*4 subunit or FGF 14-1a in mice did not affect or only partially reduced INaR, respectively (15), indicating that not all proteins capable of inducing INaR have been identified yet. In addition, proteases may be needed to tailor the proteins to be effective blocking particles (18). An alternative hypothesis of INaR genesis was recently discussed explaining INaR without the need to introduce a blocking particle (15,23,24). Modification of the Na_V_ protein is proposed to endow the *α* subunit with an altered inactivation process.

Certain toxins and mutations in Na_V_s that impair fast inactivation are natural enhancers of persistent—or late—Na^+^ currents, but they might also affect INaR (25,26,27), suggesting that at least some of their features show some interdependence. Notably, Na_V_ *α* subunits differ in their propensity to allow for INaR. Under physiological conditions, INaR has not been reported to occur in cardiomyocytes. However, Na_V_1.5, the main sodium channel subtype in the heart, gives rise to INaR, if blocking peptides are provided (*β*4 (28) and FGF13-1a (FHF2A) (22)). In fact, the resurgent current generated is particularly prominent, most likely because of the relatively slow gating kinetics of Na_V_1.5. Interestingly, FGFs were recently shown to fine-tune late currents in Na_V_1.5 (29,30). In this study, we harnessed the unusual inactivation properties of the Na_V_1.5 *Δ*KPQ mutation to interrogate the open-channel block hypothesis of the resurgent current. We also asked whether binding of the blocking particle to wild-type or mutant Na_V_1.5 has a concomitant influence on the generation of late currents and, if so, how these effects would add up to alter the sodium current trajectory of the cardiac AP.

## Materials and methods

### Plasmids and peptides

hNa_V_1.5 (NM_198056, rs763891399) was used in a pTRACER vector (Invitrogen). hNa_V_1.5 *Δ*KPQ was generated from hNa_V_1.5 using QuikChange II XL Site-Mutagenesis Kit (Agilent Technologies). C1-EGFP was obtained from Clontech. The following peptides derived from *Homo sapiens* sodium channel *β*4 (NP_777594), FGF13-1a (AAD16400), and FGF14-1a (NP_004106) according to White et al. (15) were used in this study: *β*4 (amino acids 184–196, KKLITFILKKTREK), FGF13-1a (amino acids 48–61, RVKLFGSKKRRRRR), and FGF14-1a (amino acids 50–63, KVRIFGLKKRRLRR). Neither of the full-length proteins are expressed constitutively in HEK293T cells (31). 30 mM stock solutions of peptides (PSL) were prepared in deionized water. The final concentration of 30 μM was obtained by dilution in internal solution immediately before experiments. Diluted peptides were stored at 4°C and freshly prepared before use.

### Cell culture and transfection

HEK293T cells were cultured at 37°C in humid 5% CO_2_ atmosphere in DMEM (Sigma-Aldrich) with 1-g/L glucose and supplemented with 10% fetal bovine serum (Biochrom) and 1% penicillin-streptomycin, containing 10,000 U penicillin/mL and 10 mg of streptomycin/mL (Sigma-Aldrich). 150,000 cells were plated in 3.5-cm dishes (Corning) and transfected 24 h later with 250 ng of cDNA for each hNa_V_1.5 and 100 ng of eGFP plasmid using JetPEI (Polyplus) following the manufacturer’s instructions. To minimize batch-to-batch variability, hNa_V_1.5 wild type and *Δ*KPQ constructs were transfected side by side. Transfection efficiency averaged 70%–80%, as determined by fluorescence. For patch-clamp recordings, cells displaying intermediate fluorescence intensity were selected. Electrophysiological measurements were conducted 2 days after transfection.

### Electrophysiology

Current signals were recorded in whole-cell voltage-clamp mode at room temperature (21°C ± 1°C). Recordings were sampled at 20 or 100 kHz, as stated below, and filtered at 6 kHz using a MultiClamp 700 B amplifier together with a Digidata 1320A interface and Clampex 10.3 software (all from Molecular Devices).

Access resistance in the whole-cell configuration was ≤10 M*Ω* (median = 3.6 M*Ω*) before series resistance compensation ≥75%. Electrodes were pulled from borosilicate glass (30-0057, Harvard Apparatus, or GB150F-10P, Science Products) to a resistance of 1.8–3.0 M*Ω* using a DMZ-Universal Puller (Zeitz-Instruments). The internal pipette solution contained (in mM) 135 potassium gluconate, 5 HEPES, 3 MgCl_2_, 5 EGTA, 2 Na_2_-ATP, 0.3 Na_3_-GTP, and 4 NaCl adjusted to pH 7.25 with KOH (Carl Roth). The external bath solution contained (in mM) 145 NaCl, 4 KCl, 2 CaCl_2,_ 2 MgCl_2_, 10 D-glucose, 10 HEPES, 10 TEA-Cl, adjusted to pH 7.4 with NaOH. Corrections of liquid junction potentials were not applied.

The experimental protocol was as follows: recording started 2 min after whole-cell access. The sequence of protocols was then recorded in the following order: activation with a P/4 protocol at 20 kHz, steady-state inactivation at 20 kHz without P/N correction, resurgent current with a P/5 protocol at 100 kHz, pseudo-ventricular heart AP with a P/4 protocol at 20 kHz, fast-ramp protocols, slow ramp protocol, resurgent recovery with a P/5 protocol at 100 kHz sampling frequency, and recovery of fast inactivation with a P/4 protocol at 20 kHz. Ramp recordings were leak subtracted offline.

Current-voltage (I-V) relationships were recorded with a holding potential of −100 mV for 50 ms, followed by an activation step (*V*_*activation*_) from −80 mV to +20 mV in 10 mV increments for 100 ms. The sodium conductance at different activation potentials (G_Na_) was calculated with the following equation:

$$G_{Na}=I_{Na}\;/\left(V_{activation}\;–\;V_{E}\right)$$

where *V*_*E*_ is the equilibrium potential of sodium and *I*_*Na*_ the peak inward current. For the given solutions and a recording temperature of 21°C, we calculated V_E_ = 70.7 mV. *G*_*Na*_ was normalized, and *V*_*1/2*_ and the slope factor were determined for each recording utilizing Boltzmann fit with the following equation:

$$y=\frac{A_{1}-A_{2}}{1+e^{\left(x-V1/2\right)/dx}}+A_{2}$$

where *A*_*1*_ is the lower asymptote, *A*_*2*_ is the upper asymptote corresponding to the minimal and maximal conductance, *V*_*1/2*_ is the half activation voltage, and *dx* is the slope factor. Steady-state fast inactivation was recorded with a conditioning pulse between −120 and −40 mV in 10-mV increments for 1 s and a test pulse of 0 mV. The elicited sodium current was normalized to the largest current response. A Boltzmann fit (see above) was used to approximate the data.

To measure resurgent currents, we followed the canonical protocol established previously (12). Transient sodium currents were elicited by a voltage step from −100 to 30 mV. After 12.5 ms, the membrane potential was stepped down from 0 to −90 mV (in 10-mV increments) for 300 ms. Peak currents were normalized to the peak current of the inactivation protocol (−120-mV conditioning pulse) of the respective cells. Rise and decay time constants of resurgent currents were estimated with a double exponential function using pCLAMP10 (Molecular Devices). The area under the curve (AUC) was calculated with pCLAMP10 at a time interval of 0.25 ms from the onset of the resurgent pulse to 27.75 ms. Persistent current was determined as the mean current in a 10-ms time interval 287.5 ms after the start of the resurgent pulse. In addition, we applied voltage ramps at two different slopes, 700 and 140 mV/s, to investigate persistent current using a P/N protocol.

The waveform of a heart AP was taken from a MATLAB simulation (MathWorks) of undiseased human ventricle (32) and used as voltage command. A sequence of 10 APs (each 1000 ms) was applied at a frequency of 1 Hz and averaged. To investigate the voltage dependency of the blocking mechanism by the *β*4 peptide, an additional protocol was applied. After a holding potential of −100 mV for 100 ms, test potentials from −20 to 90 mV (in 10-mV steps) were applied for 12.5 ms. With a subsequent step to −100 mV for 5 ms, displacement of the *β*4 particle was forced. Another voltage step to 0 mV revealed the fraction of available sodium currents. Additionally, we applied normalization to the currents elicited at −20 mV.

Recovery from fast inactivation was examined using a 5-ms conditioning pre-pulse from −120 to 0 mV. After varying the time intervals *Δ*t with a holding potential of −120 mV (2–200 ms), a test pulse was applied. Currents evoked by this test pulse were normalized to pre-pulse currents.

### AlphaFold modeling

The ab initio structures of the hNa_V_1.5 - h*β*4 peptide complex were generated using the deep-learning algorithms AlphaFold2 and AlphaFold Multimer version 2 (33,34). These models were run on Colab TPU servers using ColabFold v1.5.5 (35). The sequence used for the *β*4 peptide was KKLITFILKKTREK. To optimize computational efficiency, the following unstructured or disordered regions were removed from the hNa_V_1.5 sequence: R458-G657, P989-D1143, and L1960-V2016. Regions R458-G657 and P989-D1143 were replaced with short flexible linker sequences GGSSGGSSGGSSGSSS and GSSGSSGSSGSSGSSGSSGGSSGGS, respectively, to avoid structural interference with the connected structural parts. Structural modeling was performed without homology templates. MMseqs2 (36) with UniRef100 database was used for multiple sequence alignments, and each modeling run included 3–12 recycling iterations. Twenty models (four predictions per model) were generated per run. Based on the AFM confidence scores and their very similar conformation the top 15 ranked models were selected for further analysis. The peptide-protein interaction of hNa_V_1.5-*β*4 modeling showed moderate-to-good confidence, with an interface-predicted template modeling score of 0.60 and an actual interface-predicted template modeling (37) score of 0.63. The same protocol was used to predict additional complexes for Na_V_1.5 with FGF13-1a (RVKLFGSKKRRRR) and FGF14-1a (KVRIFGLKKRRLRR). However, these latter predictions produced low-confidence dimer structures (interface-predicted template modeling <0.30) and were therefore excluded from further analysis.

To minimize the energy of the side chains of the hNa_V_1.5-h*β*4 peptide models, eight consecutive rounds of structural relaxation were performed using the FoldX5 force field (38). ChimeraX (version 1.7.1) (39) was used to analyze and visualize the protein structures, including the calculation of the coulombic electrostatic potential (40). The ChimeraX matchmaker algorithm was used to compare protein structures and calculate the root-mean-square deviation (RMSD).

### Data analysis and statistics

OriginPro 2015G 64 bit was used for data analysis and visualization. Data are presented throughout as mean ± SE of the mean. For data that were not normally distributed, the geometric mean was used. Arithmetic mean ± SE of the mean was calculated on log-transformed data and the three values were back-transformed individually for graphical presentation. Statistical tests and confidence intervals are stated in each figure legend.

## Results

**Figure 1:**
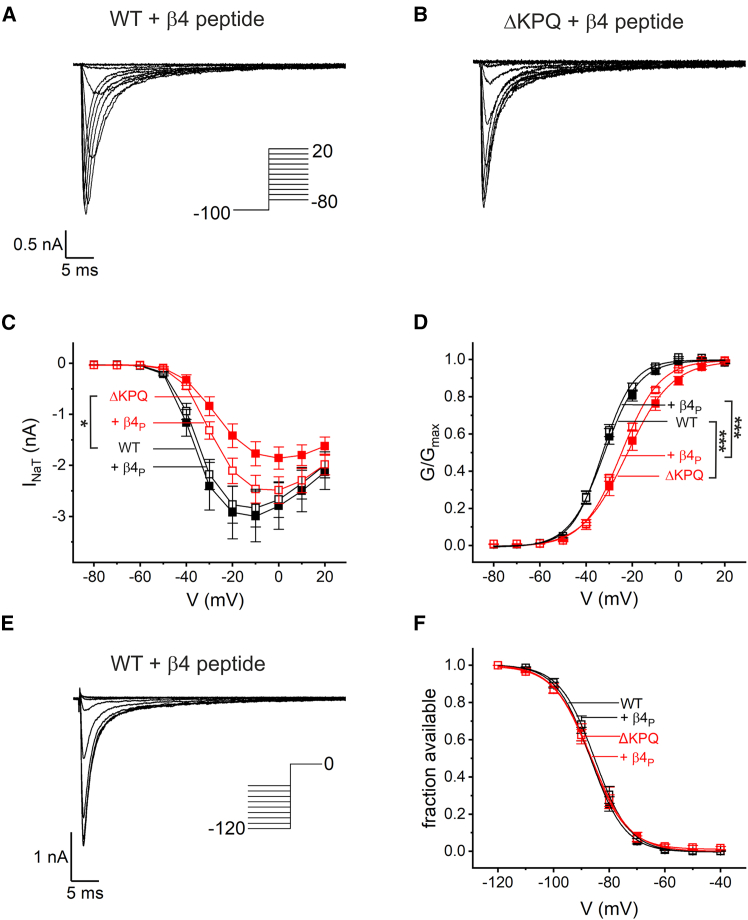
Steady-state properties of hNa_V_1.5 and *Δ*KPQ mutant channels were not vastly affected by the *β*4 peptide (*β*4_P_). Activation and inactivation properties in conjunction with the application of *β*4 peptide were investigated in whole-cell voltage-clamp experiments. HEK293T cells were transfected either with human wild-type Na_V_1.5 or with the KPQ deletion mutant. In separate experiments, the *β*4 peptide (30 μM) was included in the solution of the recording pipette for both groups. (*A* and *B*) The typical steady-state activation of wild-type hNa_V_1.5 and hNa_V_1.5 *Δ*KPQ. The voltage protocol is shown in the inset of (*A*). (*B*) Note the typical altered alignment of current peaks in mutant channels. (*C*) From recordings in (*A*) and (*B*), peak currents were plotted against the eliciting voltage step. (*D*) Each individual current-voltage relation was transformed to the underlying conductance, fitted using a Boltzmann fit and normalized to the upper asymptote. The averaged conductance is plotted against voltage. The half activation potential V_1/2_ was estimated for hNa_V_1.5 as −31.8 ± 1.2 mV, + *β*4 peptide −32.8 ± 1.0 mV, hNa_V_1.5 *Δ*KPQ −24.6 ± 0.6 mV + *β*4 peptide −32.8 ± 1.0 mV. (*E*) Steady-state fast inactivation was probed with the voltage protocol stated in the inset with a conditioning pulse of duration 1000 ms. (*F*) After normalization to the largest current, individual traces were fitted using the Boltzmann equation and plotted against voltage of the test pulse. Potentials for 50% availability were hNa_V_1.5 −86.5 ± 2.0 mV, + *β*4 peptide −85.4 ± 1.5 mV, hNa_V_1.5 *Δ*KPQ −86.1 ± 1.7 mV, + *β*4 peptide −86.8 ± 0.9 mV. Data are given as geometric mean (*C*, see section “materials and methods”) or mean ± SE (*D* and *F*). For comparison, we used a Kruskal-Wallis ANOVA (*C*) or ANOVA (*D* and *F*) with post hoc Tukey test. ^∗^*p* < 0.05, ^∗∗∗^*p* < 0.001; hNa_V_1.5 *n* = 15, + *β*4 peptide *n* = 13, hNa_V_1.5 *Δ*KPQ *n* = 13, + *β*4 peptide *n* = 24.

We investigated transient (INaT), resurgent (INaR), and persistent (INaP) sodium currents in the HEK293T expression system, combining transfection of the sodium channel *α* subunit hNa_V_1.5 or its deletion mutant *Δ*KPQ with intracellular delivery of presumed INaR-blocking particles through the whole-cell pipette. These peptides, derived from the sodium channel *β*4 subunit, FGF13-1a, or FGF14-1a, have been previously been investigated in CA3 pyramidal neurons (15), in which *β*4 and FGF14-1a peptides could generate resurgent-like currents. In the first set of experiments, we recorded the steady-state properties of hNa_V_1.5 *Δ*KPQ and wild-type channels. The data were compared to recordings made with the *β*4 peptide in the pipette (Fig. 1). The *Δ*KPQ variant decreased the peak inward currents (Fig. 1 *C*) and shifted the voltage-dependent activation toward more positive potentials (Fig. 1 *D*). In contrast, we did not observe an effect on the voltage dependency of inactivation (Fig. 1 *E* and *F*). Notably, the alignment of peak inward currents exhibited the characteristic V-shape in wild-type channels (Fig. 1 *A*), whereas, in mutant channels, the peaks were nearly vertically aligned (Fig. 1 *B*). This was attributable to faster inactivation kinetics of macroscopic currents for *Δ*KPQ, confirming previous reports (6,41). Faster inactivation rates reduce peak inward currents (32). Therefore, the reduced INaT amplitude in hNa_V_1.5 *Δ*KPQ may, at least in part, be attributed to faster inactivation. Importantly, the *β*4 peptide in the intracellular solution did not alter voltage dependence of steady-state activation and inactivation properties of wild-type or mutant channels (Fig. 1 *C*, *D* and *F*).

**Figure 2:**
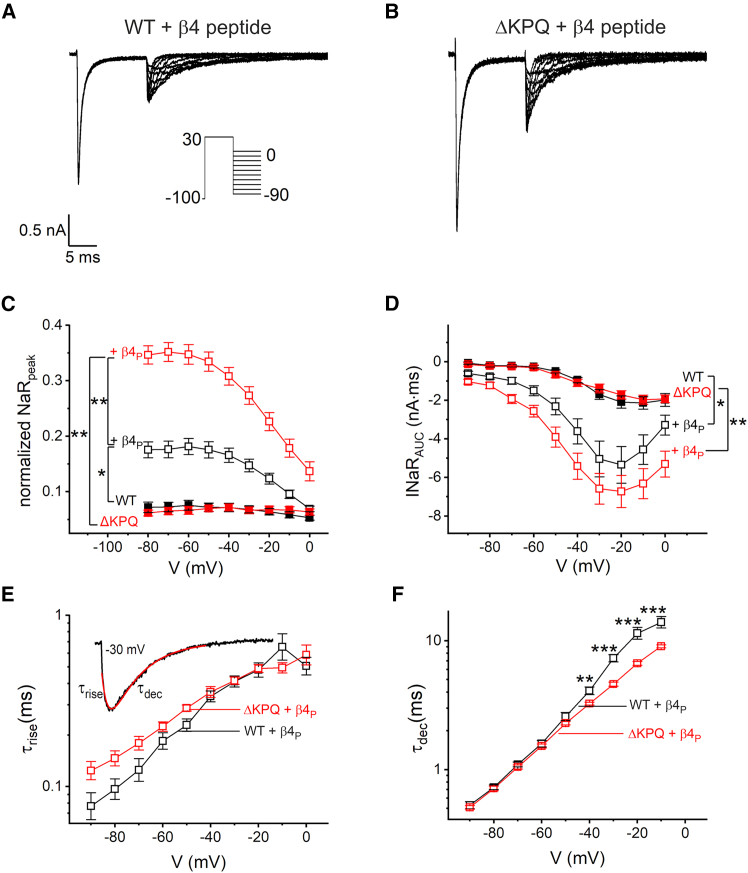
Resurgent currents elicited with *β*4 peptide (*β*4_P_) have a different time course in hNa_V_1.5 *Δ*KPQ mutant channels. HEK293T cells were transfected either with human wild-type Na_V_1.5 or with its KPQ deletion mutant. (*A*) Currents were investigated in whole-cell voltage-clamp experiments with a resurgent current protocol as depicted in the inset using a P/5 subtraction. Additionally, the *β*4 peptide (30 μM) was included in the solution of the recording pipette. (*B*) Mutant *Δ*KPQ channels typically showed a different configuration of resurgent current. (*C*) Peak currents were quantified for recordings shown in (*A*) and (*B*) and normalized to currents obtained from the inactivation protocol with a −120-mV conditioning pulse. Significances are stated for voltages ranging from −80 to −20 mV. (*D*) The integrated inward current (AUC) from the start of the resurgent voltage pulse within a time frame of 27.5 ms was quantified. For *Δ*KPQ, significances are stated for voltages >−60mV; for hNa_V_1.5, there were significant differences for voltages from −60 to −10 mV. (*E* and *F*) The current rise time and decay time of resurgent currents, respectively, were obtained from a bi-exponential fit as illustrated in the insert of (*E*) and plotted against voltage. Data are given as mean ± SE (*C* and *D*) or geometric mean (*E* and *F*; see section “materials and methods”). For comparison, we used an ANOVA (*C*), a Kruskal-Wallis ANOVA with post hoc Tukey test (*D*,) or a Mann-Whitney test (*E* and *F*).^∗^*p* < 0.05, ^∗∗^*p* < 0.01, ^∗∗∗^*p* < 0.001; hNa_V_1.5 (*n* = 15), + *β*4 peptide (*n* = 13), hNa_V_1.5 *Δ*KPQ (*n* = 13), + *β*4 peptide (*n* = 24).

To investigate the effect of the hNa_V_1.5 *Δ*KPQ mutation on INaR, a resurgent protocol was applied with a depolarization step to +30 mV for 12.5 ms followed by step repolarization (Fig. 2 *A*). With 30 μM *β*4 peptide in the pipette, this protocol yielded a substantial INaR in wild-type hNa_V_1.5 channels (Fig. 2 *A* and *C*) relative to peak transient currents. In *Δ*KPQ mutant channels normalized INaR peak currents increased by almost twofold (Fig. 2 *B* and *C*). Analysis of peak currents without the peptide is included in the graph for comparison (Fig. 2 *C*). From the depicted recordings, it becomes evident that INaR kinetics differ (Fig. 2 *A* and *B*). Detailed analysis revealed a faster current decay at more depolarized potentials in the *Δ*KPQ mutant, but no significant differences in the activation kinetics (Fig. 2 *E* and *F*). The net effect of the kinetic alterations seen in the mutant is illustrated by calculating the integrated inward current of the first 27.5 ms after the hyperpolarizing step (Fig. 2 *D*). Note that the data were not normalized to INaT peaks, because the *Δ*KPQ mutation or the peptide, by altering inactivation, simultaneously affect the peak amplitude and would skew the normalized result. The AUC was not significantly increased in hNa_V_1.5 *Δ*KPQ with *β*4 peptide compared to wild-type despite the higher INaR peak current. As a control, we included recordings without the peptide (Fig. 2 *D*) yielding only tail currents without a resurgent component (current traces not shown). It is well established that this mutation causes a significant increase in late currents (6,8). Thus, it is conceivable that the comparable integrated currents in hNa_V_1.5 *Δ*KPQ with the *β*4 peptide were attributable to an increase in INaP rather than to INaR itself. This issue will be addressed in the next section.

**Figure 3:**
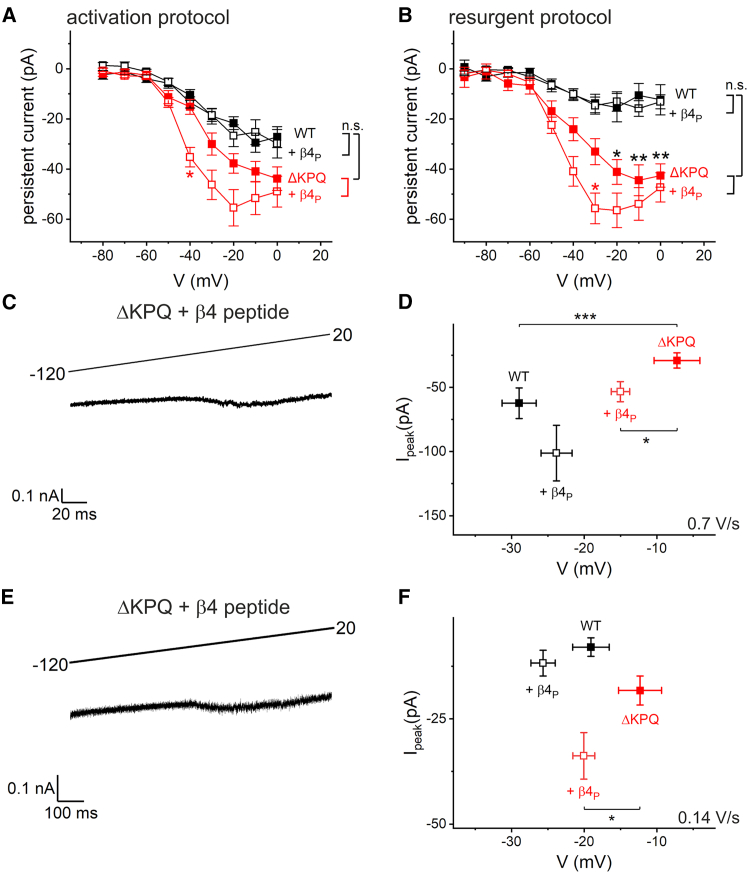
The *β*4 peptide (*β*4_P_) altered persistent currents in *Δ*KPQ channels. (*A*) Steady-state persistent currents were quantified from the activation protocol (Fig. 1*A* and *B*) at the end of the activation step for wild-type and *Δ*KPQ hNa_V_1.5 with and without the *β*4 peptide in the intracellular solution. (*B*) Additionally, steady-state persistent currents were quantified from the resurgent current protocol (Fig. 2*A* and *B*) at 287.5 ms after the repolarizing step. (*C* and *E*) INaP was also estimated from fast (0.7 V/s) and slow (0.14 V/s) voltage ramps after leak subtraction. Typical recordings are illustrated for mutant channels with *β*4 peptide in the pipette. (*D* and *F*) For quantification, the peak inward current was determined and its amplitude plotted against the voltage of occurrence. All data are given as mean ± SE. For comparison, we used an ANOVA (*A* and *B*) or MANOVA (*D* and *F*) with post hoc Tukey test. ^∗^*p* < 0.05, ^∗∗∗^*p* < 0.001; hNa_V_1.5 (*n* = 14–15), + *β*4 peptide (*n* = 13), hNa_V_1.5 *Δ*KPQ (*n* = 13), + *β*4 peptide (*n* = 22–24).

INaP was quantified (Fig. 3 *A* and *B*) from currents at the end of the activation protocol (Fig. 1 *A* and *B*) and at the end of the resurgent protocol (Fig. 2 *A* and *B*). Both protocols yielded qualitatively similar results. Under these steady-state conditions, the mutation significantly increased INaP (Fig. 3 *B*, see also Fig. 6 *D*), which is consistent with previous reports (8,9). In wild-type hNa_V_1.5, addition of the *β*4 peptide had no appreciable effect. Notably, in hNa_V_1.5 *Δ*KPQ, the peptide led to a pronounced left-shifted and increased maximum INaP. Because unbinding of the *β*4 peptide is a dynamic process indirectly related to membrane potential (16), the persistent current component was also investigated using voltage ramp protocols at different velocities dV/dt (Fig. 3 *C* and *E*). The maximum of the inward current peak was quantified together with the corresponding voltage (Fig. 3 *D* and *F*). The rather unexpected finding was that, with the ramp protocol, INaP was not significantly increased in hNa_V_1.5 *Δ*KPQ. With the fast ramp, the peak amplitude was even lower (not significant) than that of the wild-type channels and was shifted to depolarized potentials (Fig. 3 *D*). Again, the application of *β*4 peptide led to an altered voltage dependence of INaP in the mutant with a significant left-shifted peak current. In wild-type channels, no significant shift was observed (Fig. 3 *D* and *F*).

**Figure 4:**
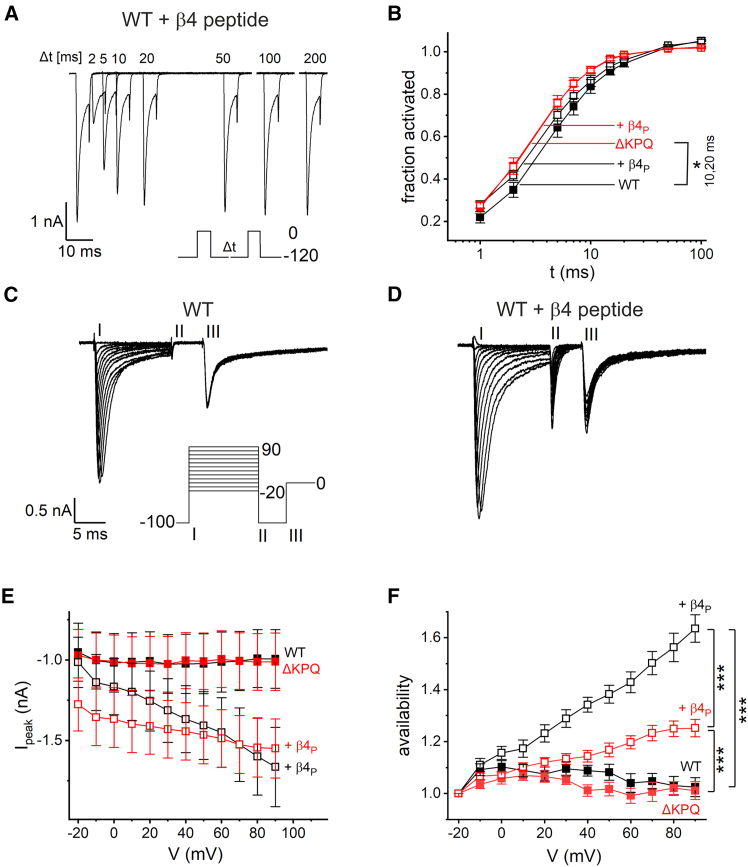
Although recovery from inactivation was not affected by the *β*4 peptide (*β*4_P_), availability was differentially increased in wild-type and *Δ*KPQ hNa_V_1.5. (*A*) Recovery from inactivation was probed with the P/4 voltage protocol depicted in the inset. After activation at 0 mV, sodium channels recovered from inactivation dependent on a pulse with variable duration *Δ*t at −80 mV. The fraction of recovered channels was probed by a second pulse to 0 mV as illustrated for this representative cell expressing hNa_V_1.5 with the *β*4 peptide in the pipette solution. (*B*) The fraction of recovery was calculated dependent on duration of *Δ*t for hNa_V_1.5, hNa_V_1.5 *Δ*KPQ and with the *β*4 peptide in the pipette. Significant differences were observed for *Δ*t = 10 and 20 ms. (*C* and *D*) Availability was tested with the voltage protocol shown in the inset. With the first pulse, *I* channels were conditioned with differing depolarization. After the 5-ms pulse *II* to −100 mV, availability was probed with a depolarizing pulse *III* to 0 mV. Note that, without the *β*4 peptide (*C*), availability is almost voltage independent, whereas, with the peptide in the pipette solution (*D*), availability increased appreciably with depolarizing conditioning pulses. (*E*) The available current was quantified for hNa_V_1.5, hNa_V_1.5 *Δ*KPQ and in addition with the *β*4 peptide. (*F*) We resolved the huge current variance of pulse *III* by normalization to the current obtained with the lowest depolarization (−20 mV) of pulse *I*. Significances are stated for 80-mV conditioning pulse only. All data are given as mean ± SE. Significances were tested using an ANOVA with a post hoc Tukey test. ^∗^*p* < 0.05, ^∗∗∗^*p* < 0.001; hNa_V_1.5 (*n* = 10–11), + *β*4 peptide (*n* = 13), hNa_V_1.5 *Δ*KPQ (*n* = 12–13), + *β*4 peptide (*n* = 23–24).

We next determined the effect of the *β*4 peptide on wild-type and *Δ*KPQ channels related to recovery from inactivation and availability. It is already known that recovery from inactivation is altered in mutant channels (8). Using a two-step protocol with a variable inter-pulse interval *Δ*t, as illustrated in Fig. 4 *A*, we estimated recovery from inactivation. Quantification of the ratio of peak currents revealed a slightly increased recovery of mutant channels at inter-pulse intervals of 10 and 20 ms (Fig. 4 *B*). With this protocol, the *β*4 peptide had only negligible effects on recovery from inactivation.

We then investigated the effect of *β*4 peptide on current availability after a given pre-pulse. Sodium channels blocked by the alternative inactivating particle do not have to recover from canonical inactivation, thereby increasing their overall availability (42). The voltage-clamp protocol was designed to activate sodium channels using a first depolarizing conditioning pulse *I* lasting 12.5 ms. Either canonical or alternative inactivation subsequently render the channels inactive. At higher depolarization voltages, alternative inactivation is favored. After a short repolarization (pulse *II*), availability was tested by stepping the membrane potential to 0 mV (pulse *III*). In the absence of the peptide in the intracellular solution, the second test pulse elicited almost uniform peak currents (Fig. 4 *C* and *E*), indicating that availability was mostly independent of the conditioning pulse. The results differed with the *β*4 peptide in the pipette solution, as illustrated in Fig. 4 *D*. Current availability increased with greater depolarizing potential of the conditioning pulse to about 1.5-fold (Fig. 4 *E*). Interestingly, the *Δ*KPQ mutant showed shallower voltage dependence in the presence of the peptide compared to wild-type channels with a similar current magnitude around +70-mV conditioning pulse. We resolved the large variance in the recorded current (Fig. 4 *E*) by normalizing it to the conditioning pulse at −20 mV (Fig. 4 *F*). For more depolarized potentials, the net gain in availability with the peptide was higher in wild-type channels (Fig. 4 *F*). The results from this voltage protocol can be summarized as follows: the *β*4 peptide has a considerable impact on channel availability, and the mutant channels have a different voltage dependency in this regard.

**Figure 5:**
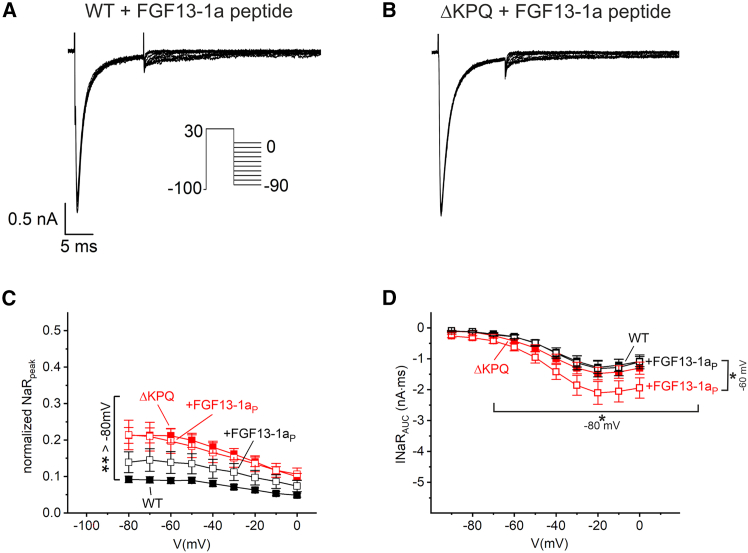
FGF13-1a peptide (FGF13-1a_P_) elicited marginal resurgent currents in hNa_V_1.5 and hNa_V_1.5 *Δ*KPQ channels. (*A* and *B*) HEK293T cells were transfected either with human Na_V_1.5 or with the *Δ*KPQ deletion mutant. Typical current traces were obtained with a resurgent current protocol as depicted in the inset using P/5 subtraction. If stated, the internal solution contained 30 μM FGF13-1a peptide. Note that control recordings without the peptide were obtained in the same recording sessions and therefore are independent of control recordings in Figs. 1, 2, and 3. (*A* and *B*) FGF13-1a peptide did not elicit prominent resurgent currents kinetics in hNa_V_1.5 or in mutant *Δ*KPQ channels. (*C*) Peak currents were quantified for recordings shown in (*A*) and (*B*) and normalized to currents obtained from the inactivation protocol with a −120-mV conditioning pulse. (*D*) In addition, without normalization, the integrated inward current (AUC) from the start of the resurgent voltage pulse within a period of 27.5 ms was quantified. Significant differences are indicated by brackets. Data are given as mean +SE (C) or geometric mean (*D*; see section “materials and methods”). Significances were tested using an ANOVA (*C*) or Kruskal-Wallis ANOVA (*D*) with a post hoc Tukey test. ^∗^*p* < 0.05; hNa_V_1.5 (*n* = 24), +FGF13-1a peptide (*n* = 20), hNa_V_1.5 *Δ*KPQ (*n* = 22), +FGF13-1a peptide (*n* = 26).

**Figure 6:**
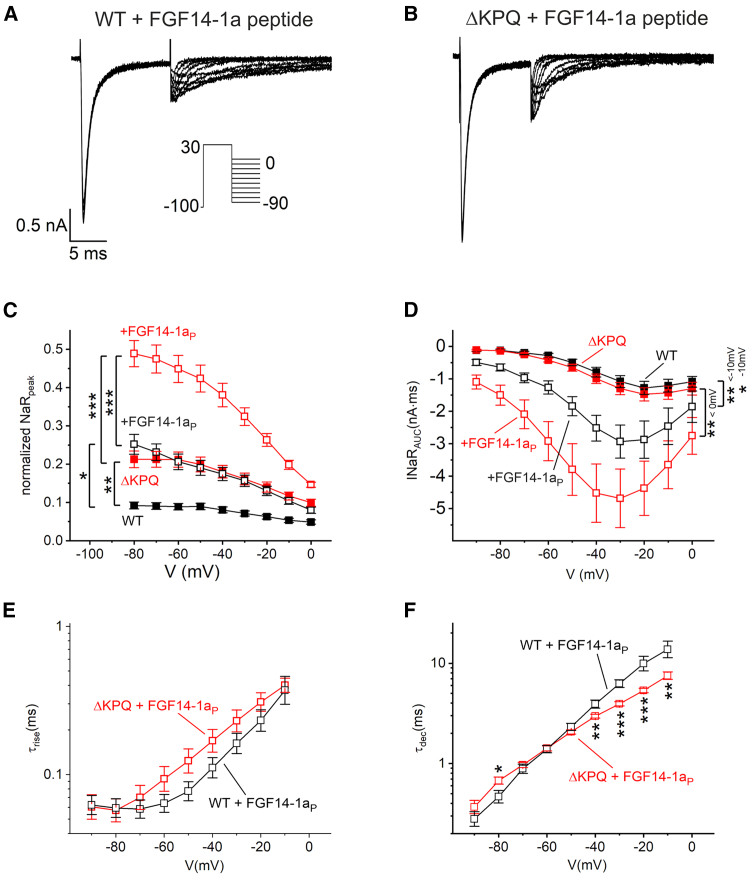
FGF14-1a (FGF14-1a_P_) peptide elicited prominent resurgent currents in hNa_V_1.5 and hNa_V_1.5 *Δ*KPQ channels. (*A* and *B*) HEK293T cells were transfected either with human Na_V_1.5 or with the *Δ*KPQ deletion mutant. Typical current traces were obtained with a resurgent current protocol as depicted in the inset using P/5 subtraction. If stated, the internal solution contained 30 μM FGF14-1a peptide. Note, control recordings without the peptide were obtained in the same recording sessions and therefore are independent of control recordings in Figs. 1, 2, and 3. (*A* and *B*) FGF14-1a peptide induced considerable resurgent currents in hNa_V_1.5 with distinct kinetics in mutant *Δ*KPQ channels. (*C*) Peak currents were quantified for recordings shown in (*A*) and (*B*) and normalized to currents obtained from the inactivation protocol with a −120-mV conditioning pulse. (*D*) In addition, without normalization, the integrated inward current (AUC) from the start of the resurgent voltage pulse within a period of 27.5 ms was quantified. Significant differences are indicated by brackets with the voltage range stated. (*E* and *F*) The current rise time and decay time of resurgent currents, respectively, was obtained from a bi-exponential fit as illustrated in Fig. 2*E* and plotted against voltage. Data are given as mean +SE (*C* and *D*) or geometric mean (*E* and *F*; see section “materials and methods”). Significances were tested using an ANOVA (*C* and *D*) or a *t*-test on log-transformed data (*E* and *F*). ^∗^*p* < 0.05, ^∗∗^*p* < 0.01, ^∗∗∗^*p* < 0.001; hNa_V_1.5 (*n* = 24), +FGF14-1a peptide (*n* = 20–21), hNa_V_1.5 *Δ*KPQ (*n* = 22), +FGF14-1a peptide (*n* = 26).

FGF13-1a and FGF14-1a were recently reported as important modifiers of late sodium currents related to hNa_V_1.5, both as peptide fragments and as full-length proteins (22,29,30). We applied the same protocols as for the *β*4 peptide to investigate INaR with 30 μM of FGF13-1a or FGF14-1a peptides in the pipette solution. Notably, the control recordings without the peptides were established in the same recording sessions and were therefore independent of the controls for comparison with the *β*4 peptide. Current traces with the FGF13-1a peptide displayed a hardly discernible fraction of INaR (Fig. 5 *A* and *B*). In contrast, the FGF14-1a peptide yielded large resurgent currents (Fig. 6 *A* and *B*). Quantification of INaR peak currents (Figs. 5 *C* and 6 *C*) and integrated INaR currents (Figs. 5 *D* and 6 *D*) substantiated this notion, with very prominent peak and integrated currents for FGF14- but not FGF13-derived peptides. Again, the FGF14-1a peptides induced INaR with distinct kinetics in hNa_V_1.5 *Δ*KPQ. Quantification of INaR activation and decay time constants revealed an altered voltage dependence of hNa_V_1.5 *Δ*KPQ current decay (Fig. 6 *F*). Kinetics were slower at −80 mV and faster at potentials more positive than −50 mV.

**Figure 7:**
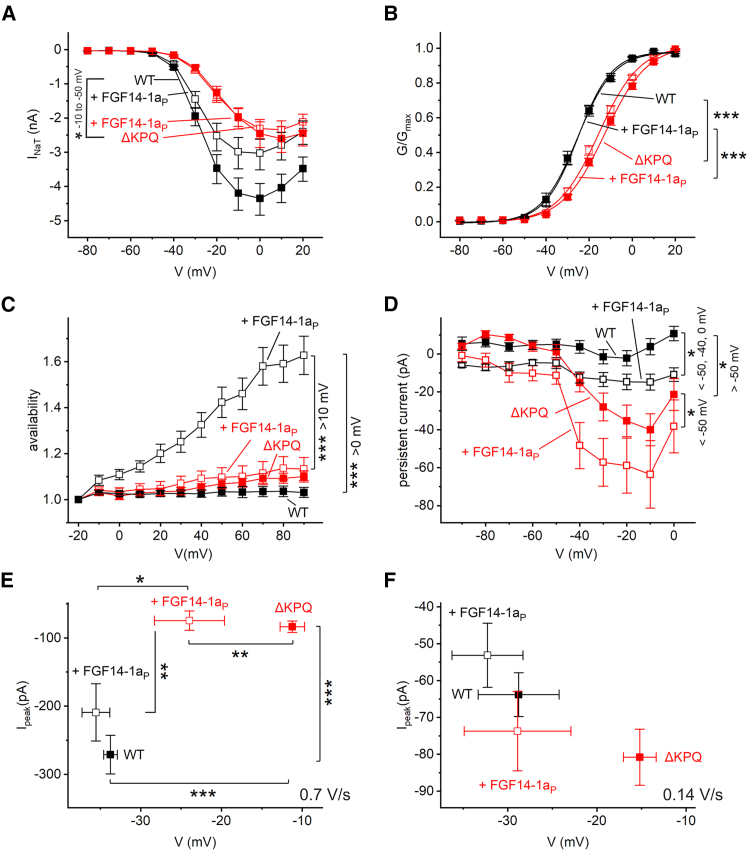
Effects of FGF14-1a (FGF14-1a_P_) peptide on transient current, availability, and persistent sodium currents. (*A*) From recordings of transient sodium currents with the activation protocol as outlined in Fig. 1*A* and *B*, peak currents were plotted against the eliciting voltage step. (*B*) The conductance was calculated from peak currents, fitted using a Boltzmann fit, normalized to the upper asymptote and plotted against voltage. The half activation potential V_1/2_ was estimated for hNa_V_1.5 −23.9 ± 0.9 mV, +FGF14-1a peptide −24.7 ± 0.9 mV, hNa_V_1.5 *Δ*KPQ −12.1 ± 1.1 mV + FGF14-1a peptide −15.4 ± 1.0 mV. (*C*) The available current was quantified for hNa_V_1.5, hNa_V_1.5 *Δ*KPQ and, in addition, with the FGF14-1a peptide from a current protocol depicted in Fig. 4*C*. Then, currents were normalized to the current obtained with the lowest depolarization of pulse *I*. (*D*–*F*) Persistent sodium currents (INaP) were quantified from (*D*) the steady-state current in the INaR protocol (Fig. 5*A* and *B*), (*E*) from fast ramps (see Fig. 3*C*), and (*F*) from slow ramps (see Fig. 3*E*) with and without FGF14-1a peptide in the intracellular solution. Data are given as geometric mean (*A*; see section “materials and methods”) or mean ± SE (*B*–*F*). Significances were tested using an ANOVA on log-transformed data (*A*), ANOVA (*B* and *C*), Kruskal-Wallis ANOVA (*D*), or MANOVA (*E* and *F*) with a post hoc Tukey test. ^∗^*p* < 0.05, ^∗∗^*p* < 0.01, ^∗∗∗^*p* < 0.001; hNa_V_1.5 (*n* = 23–25), +FGF14-1a peptide (*n* = 19–23), hNa_V_1.5 *Δ*KPQ (*n* = 20–25), +FGF14-1a peptide (*n* = 22–29).

Next, we investigated INaT and INaP using the FGF14-1a peptide in the pipette. FGF13-1a was omitted from this analysis because of its minor effect on INaR. As for the *β*4 peptide cohort (Fig. 1 *C*) with the activation protocol, absolute hNa_V_1.5 *Δ*KPQ currents were smaller than those of wild-type channels (Fig. 7 *A*), and activation was shifted to more depolarized potentials (Fig. 7 *B*). Similar to the *β*4 peptide, FGF14-1a peptide did not significantly alter INaT properties with the activation protocol. We also tested availability (Fig. 7 *C*) with the protocol outlined in Fig. 4 *C* and *D*. Given the robust effect seen in wild-type channels with FGF14-1a peptide, the lack of an effect in *Δ*KPQ mutant channels is surprising. Steady-state persistent currents were quantified at the end of the resurgent protocol. Similar to the *β*4 peptide cohort (Fig. 3 *B*), the mutation increased persistent currents (Fig. 7 *D*). In both groups, the addition of the FGF14-1a peptide further augmented INaP. In this cohort with a higher number of recordings, the *Δ*KPQ mutation showed significantly reduced persistent currents in the fast-ramp protocol (Fig. 7 *E*). This difference was not observed with the slower ramp protocol (Fig. 7 *F*). The FGF14-1a peptide shifted INaP peak to more hyperpolarized potentials with fast ramps (Fig. 7 *E*) comparable to the results observed with the *β*4 peptide (Fig. 3 *D* and *F*).

**Figure 8:**
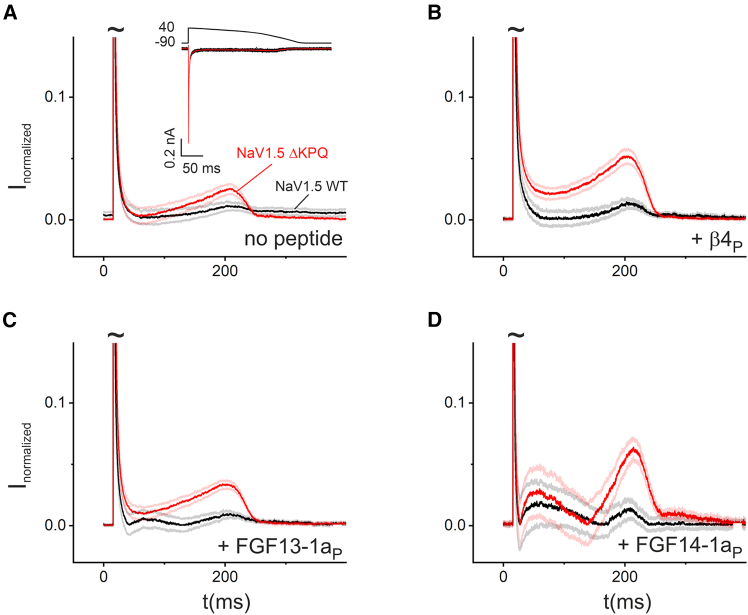
Late currents in a heart AP voltage-clamp experiment are increased in *Δ*KPQ mutant channels and further amplified with the *β*4- (*β*4_P_), FGF13-1a (FGF14-1a_P_), and FGF14-1a (FGF14-1a_P_) peptides. hNa_V_1.5 and *Δ*KPQ mutant channels were expressed in HEK293T cells. To investigate late inward currents, a ventricular cardiac AP waveform was utilized for voltage-clamp experiments with a P/4 protocol as depicted in the inset of (*A*). The waveform stated above the current trace with a total duration of 1000 ms was repeated 10 times and the average (*red trace*) was used for computing the averaged normalized current. (*A*–*D*) Recordings of hNaV1.5 (*black traces*) and hNa_V_1.5 *Δ*KPQ (*red traces*) were normalized to peak inward currents with or without 30 μM of the peptide in the pipette according to the caption (control, *β*4, FGF13-1a, FGF14-1a). Note, for better illustration, the current peak of the fast transient current is off scale (indicated by ∼). Values are given as mean ± SE (error tube). Values with nonoverlapping error tubes are considered significantly different. Control: hNa_V_1.5 (*n* = 26), hNa_V_1.5 *Δ*KPQ (*n* = 22). + *β*4 peptide: hNa_V_1.5 (*n* = 21), hNa_V_1.5 *Δ*KPQ (*n* = 23). +FGF13-1a peptide: hNa_V_1.5 (*n* = 19), hNa_V_1.5 *Δ*KPQ (*n* = 27). +FGF14-1a peptide: hNa_V_1.5 (*n* = 19), hNa_V_1.5 *Δ*KPQ (*n* = 25).

We are not aware of any studies reporting INaR in cardiac tissue, in which the resolution of small currents in response to voltage steps is challenging owing to the large capacitance-associated artifacts of these cells. Nevertheless, FGF11-14, which can enable INaR, have a fundamental impact on cardiac late sodium currents (30). Starting with the *β*4 peptide, we therefore asked how the presumed blocking particles would affect cardiac Na^+^ currents carried by wild-type hNa_V_1.5 or the *Δ*KPQ mutation. We recorded inward sodium currents from transfected HEK cells using a ventricular AP waveform as a voltage command. As shown in the inset (Fig. 8 *A*), this protocol generates a fast transient inward current, followed by a small fraction of late sodium currents in wild-type channels. For better visualization, the current traces were normalized, averaged, and displayed at a higher magnification with truncated peak currents. This representation enhances the visibility of the pathophysiologically relevant late current. Without the blocking peptide, the late currents were comparatively small in wild-type channels (Fig. 8 *A*). As expected from similar recordings (43), the mutation increased late currents with a pronounced “shoulder” before repolarization deactivated inward currents. Note that normalizing to peak currents might overestimate INaP of hNa_V_1.5 *Δ*KPQ compared to wild-type channels because of reduced INaT (Fig. 1 *C*). The addition of all the different tested peptides (Fig. 8 *B*, *C* and *D*) further increased the shoulder at the end of the AP plateau and during the initial repolarization and the plateau phase. This effect was minor in wild-type channels but was very prominent in recordings of the mutant. Interestingly, current traces, especially with the FGF14-1a peptide, appeared different from those of the *β*4 peptide with an additional peak during the plateau phase.

## Discussion

Our study on the mutant sodium channel hNa_V_1.5 *Δ*KPQ and the effects of intracellularly applied *β*4 and FGF14-1a peptides resulted in the following key findings: 1) the voltage dependence of steady-state activation and fast inactivation was not affected by the peptides. 2) Unlike another LQT3 mutation (25), hNa_V_1.5 *Δ*KPQ channels exhibited INaR with a very prominent peak and unusually fast decay kinetics. 3) The peptides increased steady-state persistent currents in mutant channels, prominently at negative potentials around −30 mV. 4) When evoked by fast voltage ramps, persistent currents of hNa_V_1.5 *Δ*KPQ were lower than those of the wild-type. The peptides shifted the peak INaP to hyperpolarized potentials. 5) The *β*4 peptide increased the availability of both wild-type hNa_V_1.5 and *Δ*KPQ channels. This effect was strongly facilitated at more depolarized potentials in wild-type channels, which was also observed with FGF14-1a peptides, but facilitation was less pronounced for *β*4 peptide or even absent for FGF14-1a peptide in mutant channels. 6) The small peptides caused strong amplification of late currents associated with the cardiac AP in the *Δ*KPQ mutant, whereas wild-type hNa_V_1.5 channels were not significantly affected, suggesting a potentially pathogenic role in hearts with dysfunctional sodium channels.

### The AlphaFold model of hNa_V_1.5 with the *β*4 peptide substantiates the proposed INaR mechanism

**Figure 9:**
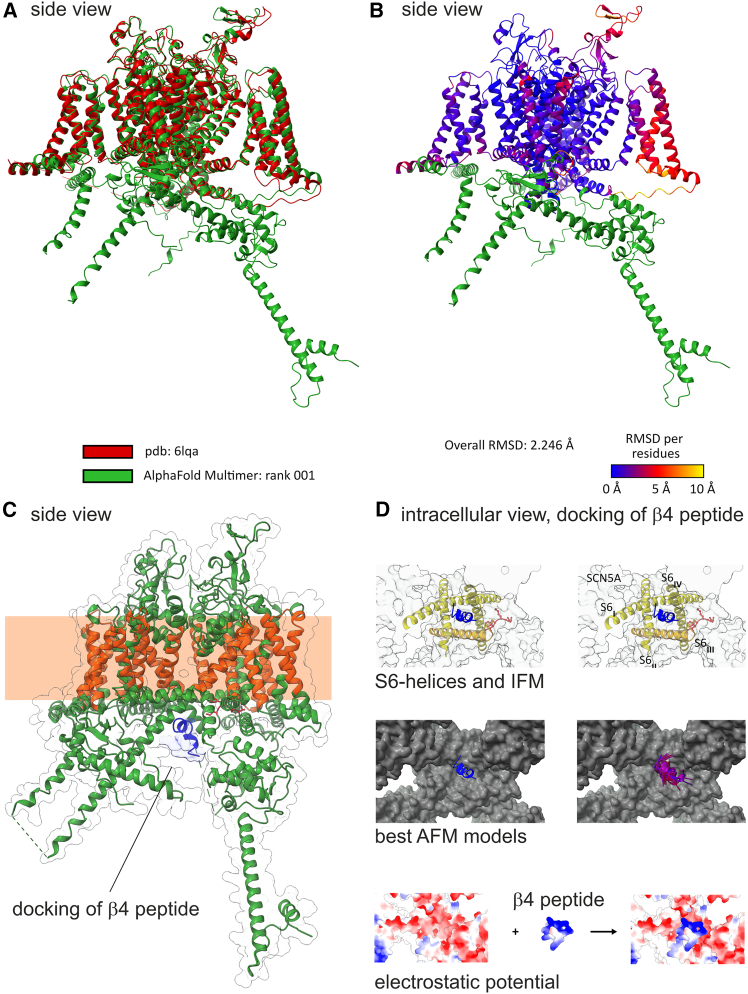
Docking of the *β*4 peptide to the human Na_V_1.5 channel. (*A*) Alignment of the cryo-EM structure of hNa_V_1.5 (44) and the best predicted structure of AlphaFold is depicted. (*B*) Color-coded root-mean-square deviation (RMSD) of both structures is displayed. Green sequences indicate residues that were not incorporated in the cryo-EM structure. (*C*) AlphaFold predicts an interaction of the *β*4 peptide (*blue*) with the hNa_V_1.5 below the intracellular gate (BIG). (*D*) The binding of the *β*4 peptide is visualized from the intracellular space depicting the bottom of hNa_V_1.5. Top: the *β*4 peptide fits directly below the pore in between the bundle crossing of the S6 helices. Middle: the model with the highest confidence locates the *β*4 peptide as shown on the left side. The 15 best models predict the location of the *β*4 peptide with only a minor deviation (*right side*). Bottom: the map of electrostatic interactions shows that the highly positive charged *β*4 peptide binds to an area of negative charges at the pore entrance.

To further investigate the interaction between the *β*4 peptide and the hNa_V_1.5 channel—which could potentially explain the observed differences in hNa_V_1.5 *Δ*KPQ—we utilized AlphaFold 2 to model the channel’s structure. In Fig. 9 *A*, we show that the AlphaFold model of hNa_V_1.5 aligns closely with a previously published cryoelectron microscopy (cryo-EM) structure (44). As indicated by blue color coding in Fig. 9 *B* and a large portion of the channel model perfectly matches the cryo-EM structure (RMSD between 0 and 2 Å). However, certain regions exhibit some deviations (red to yellow color coding) or are absent in the cryo-EM structure (green color coding). Notably, structured regions still show only moderate deviation (around 5 Å). Importantly, the AlphaFold model includes the intracellular regions of hNa_V_1.5 relevant to peptide binding, which were not resolved in published cryo-EM structures (45). Overall, we consider the quality of the AlphaFold model sufficient for generating hypotheses.

Next, we incorporated the sequence of the *β*4 peptide into the modeling process. The predicted docking position of the peptide was below the intracellular gate (BIG) of hNa_V_1.5 (Fig. 9 *C*), situated between the four S6 helices from a bottom-view perspective (Fig. 9 *D*, top panel). The confidence in this predicted location is high because the top 15 AlphaFold models placed the *β*4 peptide in nearly identical locations, with favorable peptide-protein interaction AFM scores (Fig. 9 *D*, middle panel). This position below the intracellular gating site is precisely where a docking particle was proposed to bind (46). Interestingly, the binding site is rich in negative charges, as indicated by the surface potential (Fig. 9 *D*, bottom panel). The model corroborates experimental data showing that, once the channel is open, blocking is independent of the membrane potential (16) because the binding of the peptide is facilitated electrostatically by negative charges at the mouth of the pore and the highly positively charged peptides. INaR is then generated when the influx of Na^+^ ions, driven by the electrochemical gradient, overcomes the electrostatic interaction and displaces the peptide.

**Figure 10:**
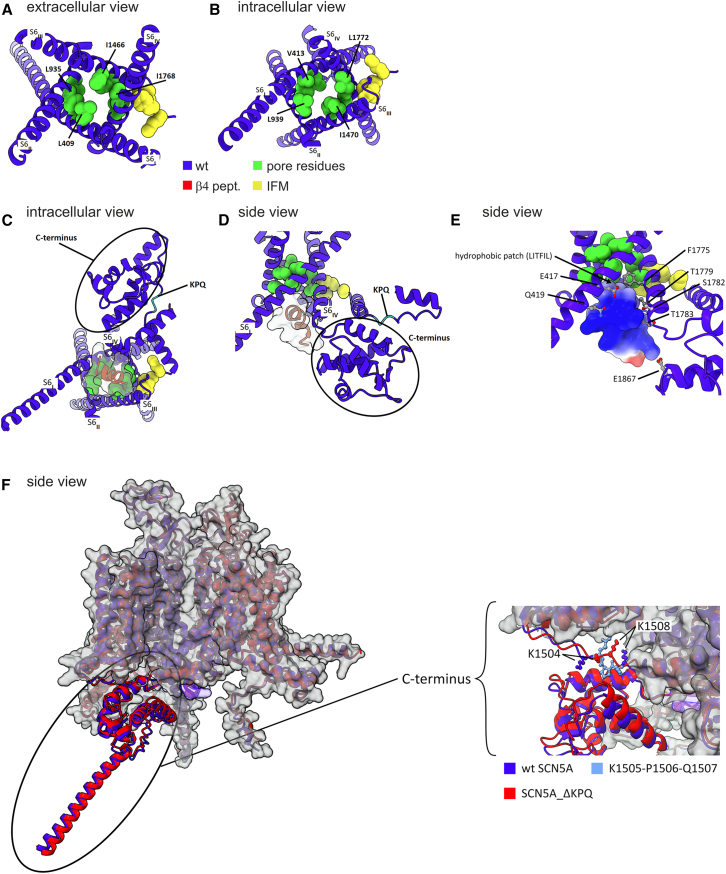
Predicted interactions. (*A*–*E*) The *β*4 peptide with the wild-type hNaᵥ1.5 channel and (*F*) the KPQ residues with the channel’s CTD. (*A* and *B*) Bulky residues lining the pore at the bundle crossing are highlighted in green and the docked IFM motif is highlighted in yellow. (*C* and *D*) Possible interactions of the docked *β*4 peptide with the pore-lining residues (*green*), S6 DIV, and especially (*E*) E1867 in the C terminus, respectively. (*F*) Predicted interaction of residues K1505 to Q1507 (*light blue*) lacking in hNa_V_1.5 *Δ*KPQ with the C terminus are illustrated. In addition, the flanking residues K1504 and K1508 are depicted. The wild-type channel (*blue*) and the channel with the deletion (*red*) are superimposed.

The canonical model of INaR posits a mutual exclusion of the blocked and fast inactivated states (46). At the predicted binding location, the *β*4 peptide cannot directly interact with the Isoleucine–Phenylalanine–Methionine (IFM) linker, which is positioned to the side of the channel in both cryo-EM structures (44,47) and the AlphaFold model (Fig. 10 *A*–*E*). Instead, the model places the peptide near the residues recently shown to close the pore after the IFM motif docks to its receptor (Fig. 10 *C*–*E*) (48). Therefore, as previously proposed (46), it is conceivable that the conformational change of the pore-lining residues induced by the docking of the IFM motif prevents the binding of the peptide. Conversely, binding of the peptide could inhibit fast inactivation by directly interacting electrostatically with E1867 of the C-terminal domain (CTD) (Fig. 10 *D* and *E*). E1867 has been shown to be in direct contact with K1504 (45), which precedes K1505-P1506-Q1507. As discussed below, the latching of the IFM linker by the CTD may not be established, thereby preventing the coupling of voltage-sensing domain 4 (VSD_IV_) activation to IFM docking (49). This mechanism is consistent with the assumption that VSD_IV_ can transition to the outward position even when the peptide is bound (50). Notably, the AlphaFold model predicts that the peptide is also in direct contact with the S6 helices, particularly S6_DI_ and S6_DIV_ (Fig. 10 *C*–*E*). This observation aligns with a recent study (51) that demonstrated the binding of the *β*4 peptide involves four residues in Na_V_1.7 located in S6_DI_, S6_DII_, and S6_DIV_.The corresponding residues are conserved in Na_V_1.5. However, in the AlphaFold model, these residues are too deep in the pore and may not be accessible to the *β*4 peptide.

Two limitations of this hypothesis are noteworthy. First, the docking of the *β*4 peptide may not represent the actual mechanism by which INaR is generated in native cells, as it remains elusive how the full-length proteins are tailored (46). Second, AlphaFold was not able to predict high-confidence models for the docking of FGF13-1a and FGF14-1a peptides at the pore of the channel. Notably, although the *β*4 peptide was predicted to adopt an *α*-helical conformation, FGF13-1a and FGF14-1a peptides were predicted to be unstructured. This may hinder AlphaFold’s prediction of the interaction between the unstructured peptides and the large hNa_V_1.5 channel protein. Furthermore, AlphaFold’s predictive power relies heavily on co-evolved residue pairs and, consequently, the interaction interface encoded in the multiple sequence alignment (MSA) (33,34). It is possible that the *β*4 peptide is an early co-evolved substrate and that FGF13-1a and FGF14-1a are later-evolved interactors. This could mean that there is insufficient information in the MSAs to generate high-confidence models of FGF13-1a and FGF14-1a peptides in combination with hNa_V_1.5. Nonetheless, based on the similarity of the electrophysiological recordings and structural features such as the accumulation of positive charges in all these peptides (15), it is reasonable to assume an analogous action of FGF14-1a peptides to bind to the negative charged pore mouth, as discussed for the *β*4 peptide.

### AlphaFold predicts impaired interaction of the IFM motif with the CTD in the hNa_V_1.5 *Δ*KPQ mutant

The hNa_V_1.5 *Δ*KPQ mutation is located in the linker between domain III (DIII) and IV (DIV) containing the IFM sequence, the key motif of the fast inactivation gate (52). Several recent studies resolved the structure of the human cardiac sodium channel with high resolution (44,47,53). *Δ*KPQ is placed in the flexible loop after a highly conserved *α* helix. This mutation is expected to shorten the DIII-DIV linker by approximately 13 Å and significantly limit the movement of the IFM motif (47). The functional consequences of the deletion that have been reported are initially accelerated fast inactivation (9,41), accelerated recovery from inactivation (8,9), and a pronounced INaP with steady-state protocols (8,9). We were able to confirm these electrophysiological findings in our study using a heterologous HEK cell expression system.

The AlphaFold model of hNa_V_1.5, including the KPQ deletion (residues 1505–1507), shows no significant structural alterations compared to the wild-type channel (Fig. 10 *F*). However, a key difference is that, in the wild-type channel, the model predicts that the *α* helix following the KPQ residues lock into a helix bundle of the EF-hand domain from the CTD (Fig. 10 *C*, *D* and *F* inset), whereas this interaction is absent in the mutant channel. This aspect of the model is strongly supported by a recent cryo-EM structure that resolved the CTD at that position (45). Based on homology modeling, previous studies have already suggested such an interaction between the IFM linker and the CTD in hNa_V_1.5 (49,54). In this mechanism, the CTD latches onto the IFM linker, preventing its binding to the receptor until VSD_IV_ is activated, which releases the physical constraint on the CTD. This latch mechanism might also explain the slower activation of VSD_IV_ compared to the other three voltage-sensing domains (49). Supporting this hypothesis, a double mutation of K1504 and K1505 to glutamate abolishes the interaction between the CTD and IFM linker (45,55). Importantly, if residues 1505–1507 are deleted (*Δ*KPQ), the AlphaFold model indicates that the shortened IFM linker cannot properly interact with the CTD. Faster inactivation, as reported for hNa_V_1.5 *Δ*KPQ (9,41) (Fig. 1 *A* vs. B), is a likely consequence. It has also been proposed that the CTD interaction stabilizes inactivation, which could explain the significantly increased late currents observed in *Δ*KPQ channels (56). Finally, it was assumed that latch impairment would cause a hyperpolarizing shift in steady-state inactivation. However, our data do not support this assumption, as we did not observe a significant shift in steady-state inactivation in our two datasets (Fig. 1 *F*, dataset of Fig. 5: hNa_V_1.5 −76.3 ± 0.6 mV, *n* = 24; hNa_V_1.5 *Δ*KPQ -78.6 ± 0.7 mV, *n* = 24). Previous findings have been contradictory, and no definitive conclusion can be drawn regarding the shift of steady-state inactivation of hNa_V_1.5 *Δ*KPQ (6,8,9,41).

### Altered INaR and INaP in hNa_V_1.5 *Δ*KPQ channels are linked to inactivation properties

Next, we addressed the unusual resurgent currents observed in hNa_V_1.5 *Δ*KPQ channels. The rapid decay of INaR can be readily attributed to the faster inactivation process (9, 41) (Fig. 1 *A* vs. 1B). The increased peak currents could be explained either by a faster unblocking of the peptides or by a higher occupancy of the blocking particle before the resurgent step. Our analysis of INaR rise time did not indicate a faster unblock (Figs. 2 *E* and 6 *E*). Given the same occupancy before the resurgent step, the integrated resurgent current should be reduced owing to the faster inactivation of *Δ*KPQ channels. In contrast, our data indicate the opposite effect. Although not statistically significant, the AUC for INaR was larger in the presence of *β*4 and FGF14-1a peptides. A similar conclusion can be drawn from the recorded availability with the peptides. Despite the reduced peak current for hNa_V_1.5 *Δ*KPQ in the activation protocol (Fig. 1 *C*), the available current at the second pulse—not normalized to peak currents—was not reduced compared to wild-type channels (Fig. 4 *E*).

Taken together, these findings strongly suggest an increased occupancy of the peptides before the repolarization step in the INaR protocol. A similar increase in INaR amplitude has been reported in other inactivation-deficient sodium channels (21,23,25,57) and in cases where toxins promote persistent currents (26,27,58). Again, a straightforward explanation is a higher open probability of the channel facilitating peptide binding. If the channel reopens, the potential docking site at the pore mouth becomes available (Fig. 10 *C*–*E*) and, through electrostatic interaction (Fig. 9 *D*), the peptide could bind. At sufficiently positive potentials, the driving force of Na^+^ ions could not overcome the electrostatic attraction and the overall occupancy increases.

Unexpectedly, we found an increased persistent current with the peptides in hNa_V_1.5 *Δ*KPQ, starting at moderate negative potentials (Figs. 3 *C*, *D* and 7 *D*) and in wild-type channels with FGF14-1a peptides (Fig. 7 *D*). At first glance, this appears counterintuitive because a blocking particle should reduce the current upon blocking or should not interfere at all. At a potential where this effect is most pronounced in *Δ*KPQ channels (−40 to −20 mV), reopening of the channel could occur either more frequently or alternatively openings could last longer because there is no evidence of a varying single-channel conductance of hNa_V_1.5 *Δ*KPQ in the literature. If the *Δ*KPQ channel reopens due to the lack of CTD-mediated stabilization of the IFM linker, the peptide is readily dispelled because of the considerable driving force for Na^+^ ions at these potentials (46). Conceivably, the peptides interfere with the fast inactivation following the unblock. The combination of the lack of interaction with the CTD at the IFM motif preceding helix and the interaction of the peptide with E1867, as discussed above, could synergistically affect fast inactivation, causing longer channel openings. This hypothesis is supported by our voltage ramp recordings. The *Δ*KPQ mutation causes a robust rightward shift and reduction in peak currents with fast voltage ramps (Fig. 3 *D* and 7 *E*). This could be explained solely by the faster inactivation observed at potentials below −10 mV (9). At potentials that are more hyperpolarized, single-channel openings are shortened. Hence, owing to the larger driving force, the macroscopic current is considerably reduced at these potentials, effectively causing a rightward shift of the peak. The same explanation is valid for the overall reduced peak currents in hNa_V_1.5 *Δ*KPQ (Fig. 1 *C*) and the resulting shift in the activation curve to more positive potentials (Fig. 1 *D*). With the ramp protocols, the application of the peptides induced a leftward shift of the peak current in the direction of the wild-type channel (Figs. 3 *D* and 7 *E*). This shift to more negative potentials could be explained by initial openings of the channel being prolonged after peptide unblocking partially mitigating the effect of faster inactivation. Importantly, data from fast-ramp protocols underscore the fact that the combination of faster inactivation and instability of fast inactivation in the mutant channel causes late currents that highly depend on the dynamics of voltage changes.

### Late currents of the *Δ*KPQ mutant are very susceptible to amplification by peptides

As shown in Fig. 7 and consistent with previous studies (8,9), the hNa_V_1.5 *Δ*KPQ mutation significantly increases late inward currents in a voltage-clamp protocol that mimics a cardiac AP waveform. Moreover, the late currents in the *Δ*KPQ channels were highly sensitive to the addition of the tested peptides; *β*4, FGF14-1a, and even FGF13-1a substantially increased the late currents. We attribute this increase largely to a INaR mechanism, given the prominent resurgent currents observed in our recordings with the *β*4 and FGF14-1a peptides. During the fast-depolarizing phase, channels accumulate in a peptide-blocked inactivated state and transition through an open state upon repolarization, further augmenting late currents. Consistent with this, simulations of cardiac APs incorporating INaR from hNa_V_1.5-F1486L mutant channels have shown increased late currents (25).

Additionally, as we have demonstrated, the peptides enhance INaP under steady-state conditions in hNa_V_1.5 *Δ*KPQ channels and even in wild-type channels when FGF14-1a peptide is present. This enhancement likely contributes to the altered current trajectory during the cardiac AP. Otherwise, it would be difficult to explain why FGF13-1a peptides, despite causing only a minimal increase in INaR (Fig. 5 *E*), still produces a considerable boost in late currents during the AP paradigm (Fig. 7 *C*).

Recently, Abrams and colleagues showed that full-length FGF13-1a expression inhibited late Na^+^ currents of mutant IQ/AA hNa_V_1.5 channels (mutation in the inactivation linker), whereas late currents of F1759A-Na_V_1.5 channels remained unchanged (29). This finding is important, because it links the action of FGF13-1a to the fast-inactivation machinery. In a follow-up study, different FGF isoforms exhibited distinct effects on hNa_V_1.5 mutant channels (30). Of note, expression of FGF13-1a (=FHF2S) reduced late currents in multi-channel recordings of hNa_V_1.5 *Δ*KPQ, whereas other splice variants did not. In addition, a peptide derived from the amino terminus of FGF12-1a, termed FixR, which proved sufficient to inhibit late currents, was proposed as a therapeutic approach. These results are supported by another study, in which expression of the shorter FGF12-1b and FGF13-1b isoforms also inhibited late currents of hNa_V_1.5 mutant channels (54).

One study disagrees with our finding that FGF13-1a peptide increases late currents (30). The apparent discrepancy might be due to one or more of the following points. 1) This study was based on full-length FGF13-1a expression, whereas we used a peptide sequence derived from the N-terminal region. The full-length proteins interact with the CTD and may affect inactivation differently than the peptides. 2) Unlike in the study by Gade et al. (54), who measured late currents only under steady-state conditions, we also used voltage ramps and heart AP waveforms, thereby demonstrating that late currents strongly differ depending on whether evoked under dynamic or steady-state conditions. 3) Normalization to INaT peak currents might yield erroneous values for late currents *if* INaT kinetics or voltage dependence are also affected by a mutation or drugs. For instance, as shown here, peak INaT currents are decreased in hNa_V_1.5 *Δ*KPQ. In addition, treatment of mutant channels with the toxin ATX-II, which amplifies late currents, simultaneously decreased INaT peak currents by more than twofold (41). Overall, the mechanism by which FGF11-14 in their numerous isoforms mediate INaR versus affecting INaP are not yet well understood (46).

Proteins such as the *β*4 subunit or FGF13 are expressed in cardiac tissue (59,60). Recently, expression of different FGFs was reported from human iPSC-derived cardiomyocytes including LQT3 patients carrying the *Δ*KPQ mutation (30). The expression pattern included FGF14, albeit at a low level. Furthermore, a FGF13-1a derived peptide (FHF2A_2-18_) mediates an open-channel block of hNa_V_1.5 (61). Likewise, heterologous expression of FGF13-1a (FHF2A) with hNa_V_1.5 in HEK293 cells was found to induce a small INaR (22). Although INaR has not yet been reported in native cardiac cells, the situation might be different in the diseased heart, particularly in patients carrying the *Δ*KPQ mutation. In contrast to late currents from wild-type hNa_V_1.5, which are hardly affected, our study revealed a strong impact of FGF13-1a and FGF14-1a derived peptides on late currents from *Δ*KPQ mutant channels. Assuming a scenario in which cardiac stress or damage causes the FGF expression levels to rise (62,63), putative proteases could yield peptides that cause pathologically enhanced INaR. This would increase the risk of severe cardiac arrhythmias in patients with long QT syndromes.

## Conclusions

Our recordings of the inactivation-deficient hNa_V_1.5 *Δ*KPQ channel using synthetic peptides as pore blockers revealed intriguing differences related to persistent and resurgent sodium currents compared to wild-type channels. We further corroborated these findings using an AlphaFold model of hNa_V_1.5—including the artificial *β*4 peptide—to elucidate the molecular interplay between the inactivation mechanism and peptide binding that leads to resurgent sodium currents. The model aligns closely with previous cryo-EM structures and homology modeling studies, reinforcing its validity. It resolves key aspects of the previously proposed pore block model (64) and refines the concept of mutual exclusion between pore block and fast inactivation by highlighting an interaction between the peptide and the CTD of hNa_V_1.5. This suggests an intimate interplay between the peptide-mediated pore block and the fast-inactivation machinery. This model could serve as a framework for further refining our understanding of the molecular mechanisms of INaR. Additionally, our data indicate a substantial increase in late currents in hNa_V_1.5 *Δ*KPQ channels if a blocking particle is present in cardiac cells, highlighting a potential pathogenic mechanism in cardiac arrhythmias.

## Data availability

The datasets used and/or analyzed during the current study are available from the corresponding authors on reasonable request.

## Acknowledgments

We are grateful to Iwona Izydorczyk for technical assistance. We thank Stephanie Hartmann, Kerstin Völkl, Tilmann Volk, and Michael Wagner for initial experimental support. We gratefully acknowledge Marie Liselott Mehlfeldt for her thoughtful and meticulous proofreading. This work was supported by the Deutsche Forschungsgemeinschaft (DFG, German Research Foundation) – Project number HU 2358/1-1 (granted to T.H.). The present work was performed in (partial) fulfillment of the requirements for obtaining the degree Dr. med.

## Author contributions

M.R., formal analysis, investigation, and visualization; M.W., formal analysis, investigation, and visualization; P.J.W., formal analysis, visualization, and writing – review & editing; K.F., formal analysis and investigation; S.H., formal analysis and supervision; A.L., conceptualization and writing – review & editing; C.A., conceptualization and writing – review & editing; S.D., conceptualization, formal analysis, investigation, methodology, and writing – review & editing; T.H., conceptualization, formal analysis, funding acquisition, project administration, supervision, visualization, and writing – original draft.

## Declaration of interests

A.L. receives consulting fees from Grünenthal on a project unrelated to the one presented here.
